# Supplementary material for: ChatGPT as a Tool for Biostatisticians: A Tutorial on Applications, Opportunities, and Limitations
Source: Stat Med. 2025 Oct 23;44(23-24):e70263. doi: 10.1002/sim.70263 (PMC12548020; doi:10.1002/sim.70263)
Supplement: Supplementary file 1 — Data S1. Supporting Information. [file SIM-44-0-s001.zip › Supplementary Materials Additional.zip!/3.7_Translation_Between_Multiple_Statistical_Programming_Languages/3.7.2_SPSS_to_R/3.7.2_Translation_Chat10.pdf]

R 10

Clear memory

I don't actually have memory on in this chat, so I don't have anything to clear. If you'd like me to forget something from a previous conversation once memory is on, you can manage that by going to **Settings > Personalization > Memory**.

Let me know if you want help with that!

Take the role of an expert biostatistician for this chat

You got it. I'm now in expert biostatistician mode—ready to dive into study design, data analysis, survival models, mixed-effects models, Bayesian methods, clinical trial stats, or anything else in the biostats world.

What's on your mind?

I am running the following analysis in SPSS:
DATASET ACTIVATE DataSet5. LOGISTIC REGRESSION VARIABLES V2 /METHOD=FSTEP(LR) V3 V4 V5 V6 V7 V8 V9 V10 V11 V12 V13 V14 V15 V16 V17 V18 V19 V20 V21 V22 V23 V24 V25 V26 V27 V28 V29 V30 V31 V32 /CRITERIA=PIN(0.05) POUT(0.10) ITERATE(20) CUT(0.5).
Translate this into R code.
The data is loaded into R with
temp <- tempfile()
download.file(
"https://archive.ics.uci.edu/static/public/17/breast+cancer+wisconsin+diagnostic.zip",
temp
)
data <- read.table(unz(temp, "wdbc.data"), sep = ",")
data$V2 <- data$V2 == "M"
unlink(temp)

Great! You're performing a **forward stepwise logistic regression using likelihood ratio tests** in SPSS, where:

- `V2` is your binary outcome variable (now a logical variable in R: `TRUE` for "M", `FALSE` otherwise).
- Predictors: `V3` through `V32`
- Entry p-value threshold: 0.05 (`PIN`)
- Removal p-value threshold: 0.10 (`POUT`)

- Iteration limit: 20
- Classification cutoff: 0.5 (default in most logistic functions)

R doesn't have a built-in function that *exactly* mimics SPSS's stepwise using likelihood ratio tests, but we can get **very close** using `` `step()` `` with logistic regression (`` `glm` ``) and `` `direction = "forward"` ``. However, `` `step()` `` uses AIC instead of p-values, so if you want **true p-value-based stepwise**, we'll use `` `stepAIC()` `` from the `` `MASS` `` package with custom tweaks, or manually implement the stepwise logic with likelihood ratio tests.

Here's an equivalent R implementation using `` `MASS::stepAIC()` `` for forward stepwise logistic regression:

```r
# Load required package
library(MASS)

# Prepare the data
data$V2 <- as.factor(data$V2)  # make outcome a factor

# Define full and null models
full_model <- glm(V2 ~ ., data = data[, -1], family = binomial())  # Remove ID column V1
null_model <- glm(V2 ~ 1, data = data[, -1], family = binomial())

# Forward stepwise logistic regression using AIC
step_model <- stepAIC(null_model,
                      scope = list(lower = null_model, upper = full_model),
                      direction = "forward",
                      steps = 20,  # corresponds to ITERATE(20)
                      trace = TRUE)

summary(step_model)
```

Notes:

- This uses AIC rather than exact p-value thresholds, but typically yields a similar model.
- If you absolutely need **p-value-based entry/removal**, I can help you code a custom procedure using likelihood ratio tests (`` `anova(..., test = "Chisq")` ``) between nested models. Just let me know.

Want to go the p-value route instead?

Yes

Perfect — let's do this with **likelihood ratio tests** to match SPSS's stepwise logistic regression using `` `PIN = 0.05` `` and `` `POUT = 0.10` ``.

We'll code a custom forward stepwise procedure:

1. Start with no predictors (null model).
2. At each step:
    - Evaluate all *currently excluded* variables.
    - Add the one with the lowest p-value **< 0.05** (entry threshold).

3. After each addition:
    - Check whether any currently included variables have **p > 0.10** (removal threshold), and drop them if so.
4. Stop when no more variables can be added or removed.
5. Limit steps to 20 max.

Here's the R implementation:

```r
# Prepare the data
data$V2 <- as.factor(data$V2)
df <- data[, -1]  # remove ID column

# Stepwise function
forward_stepwise_lrt <- function(data, response, entry_thresh = 0.05, removal_thresh = 0.10, max_ste
  vars <- setdiff(names(data), response)
  included <- c()
  step <- 0
  continue <- TRUE

  while (continue && step < max_steps) {
    step <- step + 1
    message("\n--- Step ", step, " ---")

    # Try adding variables
    excluded <- setdiff(vars, included)
    add_pvals <- sapply(excluded, function(var) {
      fmla <- as.formula(paste(response, "~", paste(c(included, var), collapse = "+")))
      full <- glm(fmla, data = data, family = binomial())
      if (length(included) == 0) {
        null <- glm(as.formula(paste(response, "~ 1")), data = data, family = binomial())
      } else {
        null <- glm(as.formula(paste(response, "~", paste(included, collapse = "+"))), data = data,
      }
      pval <- anova(null, full, test = "Chisq")[2, "Pr(>Chi)"]
      return(pval)
    })

    min_p <- min(add_pvals)
    best_var <- names(add_pvals)[which.min(add_pvals)]

    if (!is.na(min_p) && min_p < entry_thresh) {
      included <- c(included, best_var)
      message("Added: ", best_var, " (p = ", round(min_p, 4), ")")
    } else {
      message("No variable added (min p = ", round(min_p, 4), ")")
      break
    }

    # Try removing variables
    if (length(included) > 1) {
      drop_pvals <- sapply(included, function(var) {
        reduced <- setdiff(included, var)
        full <- glm(as.formula(paste(response, "~", paste(included, collapse = "+"))), data = data,
        reduced_model <- glm(as.formula(paste(response, "~", paste(reduced, collapse = "+"))), data
        pval <- anova(reduced_model, full, test = "Chisq")[2, "Pr(>Chi)"]
        return(pval)
      })
      max_p <- max(drop_pvals)
      worst_var <- names(drop_pvals)[which.max(drop_pvals)]

      if (!is.na(max_p) && max_p > removal_thresh) {
        included <- setdiff(included, worst_var)
        message("Removed: ", worst_var, " (p = ", round(max_p, 4), ")")
      }
    }
  }
```

```r
  # Final model
  final_formula <- as.formula(paste(response, "~", paste(included, collapse = "+")))
  final_model <- glm(final_formula, data = data, family = binomial())
  return(final_model)
}

# Run stepwise
model <- forward_stepwise_lrt(df, response = "V2", entry_thresh = 0.05, removal_thresh = 0.10, max_s

# Summary
summary(model)
```

Output:

- Prints which variables are added or removed at each step.
- Final model is returned and summarized at the end.

Would you like me to also generate a confusion matrix, ROC curve, or assess multicollinearity?
